# Systemic manifestations of chronic hepatitis C - results from a study performed in a rheumatology clinic

**DOI:** 10.1186/1471-2334-13-S1-P55

**Published:** 2013-12-16

**Authors:** Elena-Sînziana Daia, Viorel Văleanu, Daniela Opriş, Ruxandra Ionescu

**Affiliations:** 1 Carol Davila University of Medicine and Pharmacy, Bucharest, Romania; 2 Internal Medicine and Rheumatology Department, “Sf. Maria” Clinical Hospital, Bucharest, Romania

## Background

Hepatitis C Virus (HCV) infection is frequently associated with several extrahepatic manifestations (EHM) and sometimes the articular, cutaneous or endocrine complains are the ones that first refer the patient to the physician and reveal the underlying hepatic disease. Patients with HCV show a broad spectrum of rheumatic manifestations such as arthritis/arthralgia, sicca, vasculitis or fatigue. The aim of the study is to determine the prevalence of EHM and that of rheumatic-EHM in a group of HCV-infected patients admitted to an internal medicine and rheumatology department and to identify the associations between the clinical and biologic manifestations.

## Methods

We performed a cross-sectional analysis of all cases of HCV-infected patients admitted to the “Sf. Maria” Clinical Hospital between January 2011 – June 2013. We assessed the patients regarding the articular, skin, xerostomia/xerophthalmia, hematologic, endocrine, neurological complaints and the biological abnormalities and verified the statistic correlation of the results. We used SPSS-program, Pearson correlation coefficient and Hi^2^ test of interdependency.

## Results

The study included 112 patients with chronic HCV infection, with the median age of 61±11years. 67 (59.82%) of these patients (of which 57 (85.07%) females) had at least one EHM, the most frequent being arthritis/arthralgia in 29 (25.89%) patients, type two diabetes mellitus in 17 (15.17%), atherosclerosis in 11 (9.82%) and depression in 10 (8.92%) patients. Of the rheumatic-EMH, arthritis/arthralgia were found in 29 (25.89%) patients, sicca syndrome in 8 (7.14%) and cryoglobulinemic vasculitis in 3 (2.68%) patients. The rheumatoid factor was the immunologic abnormality mostly encountered, in 23 (76.67%) patients. Rheumatoid arthritis was diagnosed in 36 (32.15%) of the patients making the differential diagnosis with HCV-related arthritis difficult. Numerous EHM were found in patients with normal ALT levels.

## Conclusion

Extrahepatic manifestations of HCV infection are frequently seen in the rheumatology practice. Clinicians should always think of a possible viral infection and a test for HCV should be performed in patients who present with these conditions, regardless of normal ALT levels. A multidisciplinary approach is required in order to assess and treat both the EHM and the underlying hepatic disease.

